# The mortality risk in patients with early onset colorectal cancer: the role of comorbidities

**DOI:** 10.3389/fonc.2023.1139925

**Published:** 2023-04-14

**Authors:** Shou-Chun Yu, Yow-Ling Shiue, Yu-Cih Wu, Jhi-Joung Wang, Kuang-Ming Liao, Chung-Han Ho

**Affiliations:** ^1^ Institute of Biomedical Sciences, National Sun Yat-sen University, Kaohsiung, Taiwan; ^2^ Department of Medical Research, Chi-Mei Medical Center, Chiali, Tainan, Taiwan; ^3^ Department of Medical Research, Chi Mei Medical Center, Tainan, Taiwan; ^4^ Department of Anesthesiology, Tri-Service General Hospital and National Defense Medical Center, Taipei, Taiwan; ^5^ Department of Internal Medicine, Chi Mei Medical Center, Chiali, Tainan, Taiwan; ^6^ Department of Information Management, Southern Taiwan University of Science and Technology, Tainan, Taiwan; ^7^ Cancer Center, Taipei Municipal Wanfang Hospital, Taipei Medical University, Taipei, Taiwan

**Keywords:** early-onset colorectal cancer, mortality risk, comorbidities, Taiwan cancer registry, real-world database (RWD)

## Abstract

The global incidence of early-onset colorectal cancer (EO-CRC) is increasing. Although the mortality rate is relatively stable, some comorbidities have been associated with a higher mortality rate. This study estimated the mortality risk in patients with EO-CRC with various comorbidities using real-world data to identify the high-risk group using Cox proportional regression for overall and cancer-specific mortality. The incidence rate of EO-CRC significantly increased from 6.04 per 100,000 population in 2007 to 12.97 per 100,000 population in 2017. The five-year overall mortality rate was 101.50 per 1000 person year and the cancer-specific mortality rate was 94.12 per 1000 person year. Patients with cerebrovascular disease (CVD) had a higher mortality risk (hazard ratio (HR): 1.68; 95% confidence interval (CI): 1.25-2.28; p=0.0007). After subgroup analyses based on age, sex, clinical stage, and treatment type, patients with CVD had a higher overall mortality risk compared to non-CVD patients, except for patients undergoing surgery and chemotherapy. Patients with chronic kidney disease had a higher mortality risk in the early clinical stages (HR: 2.31; 95% CI: 1.08-4.96; p=0.0138). Patients who underwent radiotherapy had a higher overall mortality risk (HR: 1.38; 95% CI: 1.04-1.85; p=0.0285) than those without liver disease. Identifying specific comorbidity mortality risks in patients with EO-CRC allows for risk stratification when screening target groups and may lower disease mortality.

## 1 Introduction

Cancer is one of the leading causes of death worldwide, and colorectal cancer (CRC) was the second leading cause of cancer death in 2020 (1). The age-standardized mortality rate for colorectal cancer is approximately 8.9 per 100,000 population; however, patients with CRC aged under 50 years of age have a higher survival rate than those over 50 years of age (2). The mortality rate of early-onset colorectal cancer (EO-CRC) increased by approximately 1.3% per year from 2008 to 2017, except for patients aged 20-29 years, for whom it remained stable (3).

The incidence of CRC decreased in patients aged 50 years or older, though the incidence increased in young adults. In patients less than 50 years old, the incidences of localized, regional, and distant CRC increased. The incidence of CRC is predicted to increase by 90.0% by 2030 in patients aged 20-34 years (4). In 2021, the US Preventive Services Task Force reported that screening for CRC in adults aged 50-75 years has a substantial net benefit, and screening for CRC in adults aged 45-49 years has a moderate net benefit (5). Before 2021, patients aged 45-49 years did not undergo regular screening for CRC. The incidence of EO-CRC is also increasing in other countries, including France (6), Canada (7), Australia (8), Japan, South Korea, and Taiwan (9). The incidence of EO-CRC increased by 3.9% per year in Taiwan and 6.0% per year in South Korea (9).

In Taiwan, the increasing incidence of CRC is attributed to the implementation of population-based screening programs and westernized dietary lifestyle over the past three decades (10, 11). The Taiwanese government offered a nationwide screening program using a fecal immunochemical test for individuals aged 50 to 75 years (12), though the failure to screen patients younger than 50 years may have resulted in an increased rate of advanced CRC in young patients. Potential risk factors associated with EO-CRC include diet, a sedentary lifestyle, smoking, and alcohol consumption (7, 13–15). Few studies regarding the mortality risk factors in patients with EO-CRC that use real-world data have been reported; studies using real-world data typically report the patient’s age and clinical stage of the disease (16, 17). However, the comorbidities of patients with CRC may also affect the treatment, prognosis, and survival (18–20). This study aimed to understand the role of comorbidities in the mortality risk of patients with EO-CRC using population-based, real-world data.

## 2 Materials and methods

### 2.1 Data sources

Data from patients with CRC registered in the Taiwan Cancer Registry (TCR) and the National Health Insurance research database were used in this study. The TCR database was established in 1979 with a high-quality, precise diagnosis, and treatment items to track the cancer incidence and mortality rates in Taiwan. Long-form datasets for cancer in the colon and rectum, oral cavity and pharynx (except nasopharynx), liver, lung, breast, and cervix were added to the TCR in 2002, and prostate cancer was added in 2007 (21, 22).

To obtain data regarding comorbidities and medical history, administrative claims from Taiwan’s National Health Insurance program, which covers all inpatient and outpatient health services, were also used in this study. For research purposes, Taiwan’s Health and Welfare Data Science Center (HWDC) integrated the population database, which was linked from different health-related datasets, and managed the application to avoid violations of personal information protection.

### 2.2 Study population

**Figure 1:**
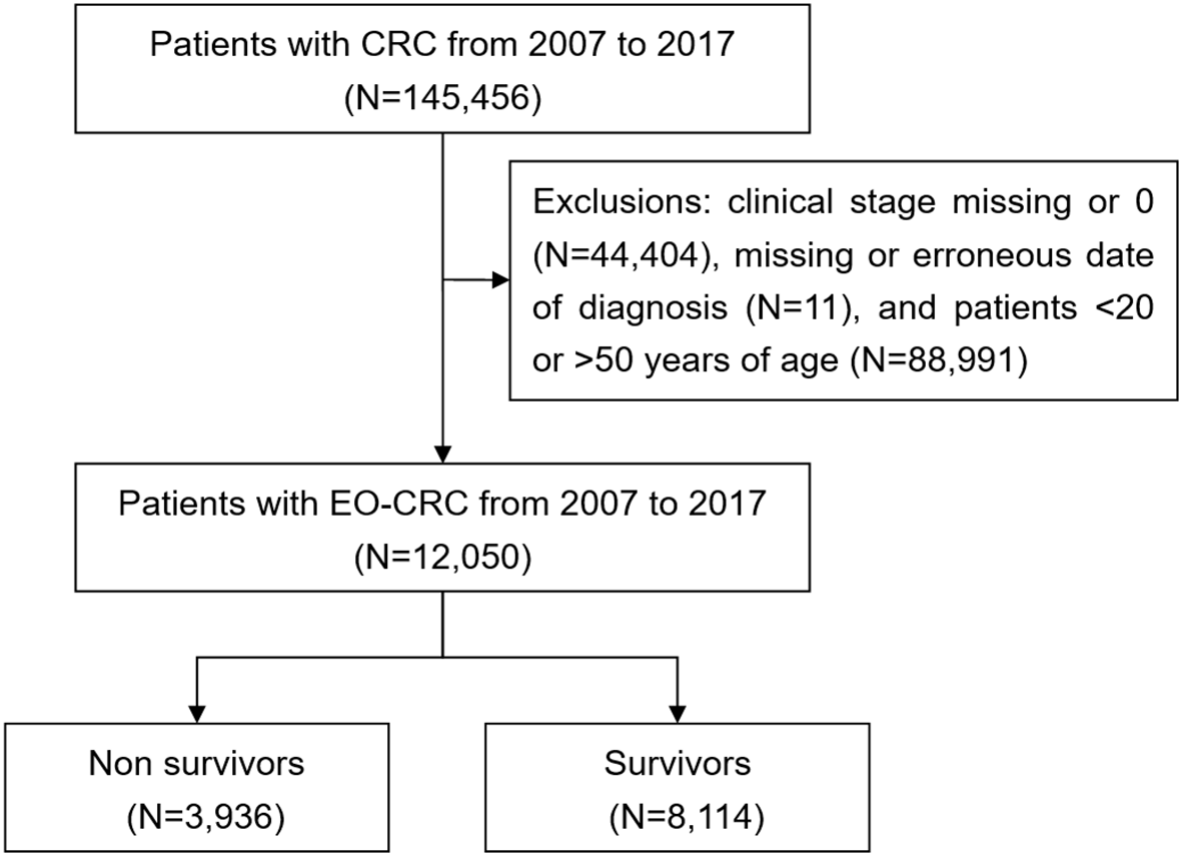
Patient selection flowchart.

Patients with CRC were selected from the TCR using the International Classification of Diseases for Oncology, third edition (ICD-O-3) classification for CRC (ICD-O-3: C18-C20) from January 2007 to December 2017. All patients were followed up until December 31, 2017. The maximum follow-up period was five years after the date of CRC diagnosis. EO-CRC is defined as CRC in patients less than 50 years of age. Therefore, only patients less than 50 years old were included in this study. Patients with a history of cancer (before the diagnosis of CRC) and those with missing data were excluded from the study (**Figure 1**).

### 2.3 Measurements

The patients’ baseline characteristics, including age, sex, clinical stage, treatment type, Charlson’s comorbidity index (CCI) score, and comorbidities, were recorded. The patients were classified into three age groups: <30 years, 30-39 years, and 40-49 years. The CCI score was calculated as previously described (23, 24). The patients were divided into three disease severity groups based on CCI score: 0, 1-2, and ≥3. Comorbidities, including congestive heart failure, peripheral vascular disease, cerebrovascular disease, chronic pulmonary disease, liver disease, chronic kidney disease, diabetes mellitus, hyperlipidemia, and hypertension, were identified using the International Classification of Diseases, Ninth Revision, Clinical Modification or the International Classification of Diseases, Tenth Revision, Clinical Modification diagnostic codes (**Table S1**). The comorbidities were included in the study if they were noted during hospitalization or three or more outpatient visits within one year before the cancer diagnosis.

### 2.4 Outcomes

The primary outcome of this study was overall mortality, which was identified as death for any reason. In addition, cancer-specific mortality was used to estimate the risk of mortality due to cancer. Mortality was defined using Taiwan’s cause-of-death database.

As different comorbidities may affect the mortality risk, the overall and cancer-specific mortality analyses of patients based on age, sex, clinical stage, and treatment type were estimated for each comorbidity.

### 2.5 Statistical analysis

The incidence of EO-CRC was estimated as the number of patients aged 20-49 years with CRC divided by the total population of 20-49-year-olds that year. The trend of the mortality rate was defined as the number of patients with EO-CRC who died within one year divided by the total number of patients with EO-CRC that year. Categorical variables, including age group, sex, clinical stage, treatment type, CCI score classification, and comorbidities, are presented as numbers and percentages. Pearson’s chi-square test was used to compare the differences in the categorical variables between patients with EO-CRC who died within five years of the diagnosis and those who survived. Continuous variables, such as age, CCI score, and time to follow-up are presented as mean ± standard deviation. Student’s t-test was used to compare the differences in the continuous variables between patients who died within five years of the diagnosis and those who survived. The Cox proportional regression model was used to estimate the overall and cancer-specific relative risk of mortality. A stratified analysis of age, sex, clinical stage, and treatment type was conducted. All p values are two-tailed, and statistical significance was set at p<0.05. All statistical analyses were conducted using the SAS software (version 9.4; SAS Institute, Inc., Cary, NC, USA).

## 3 Results

**Figure 2:**
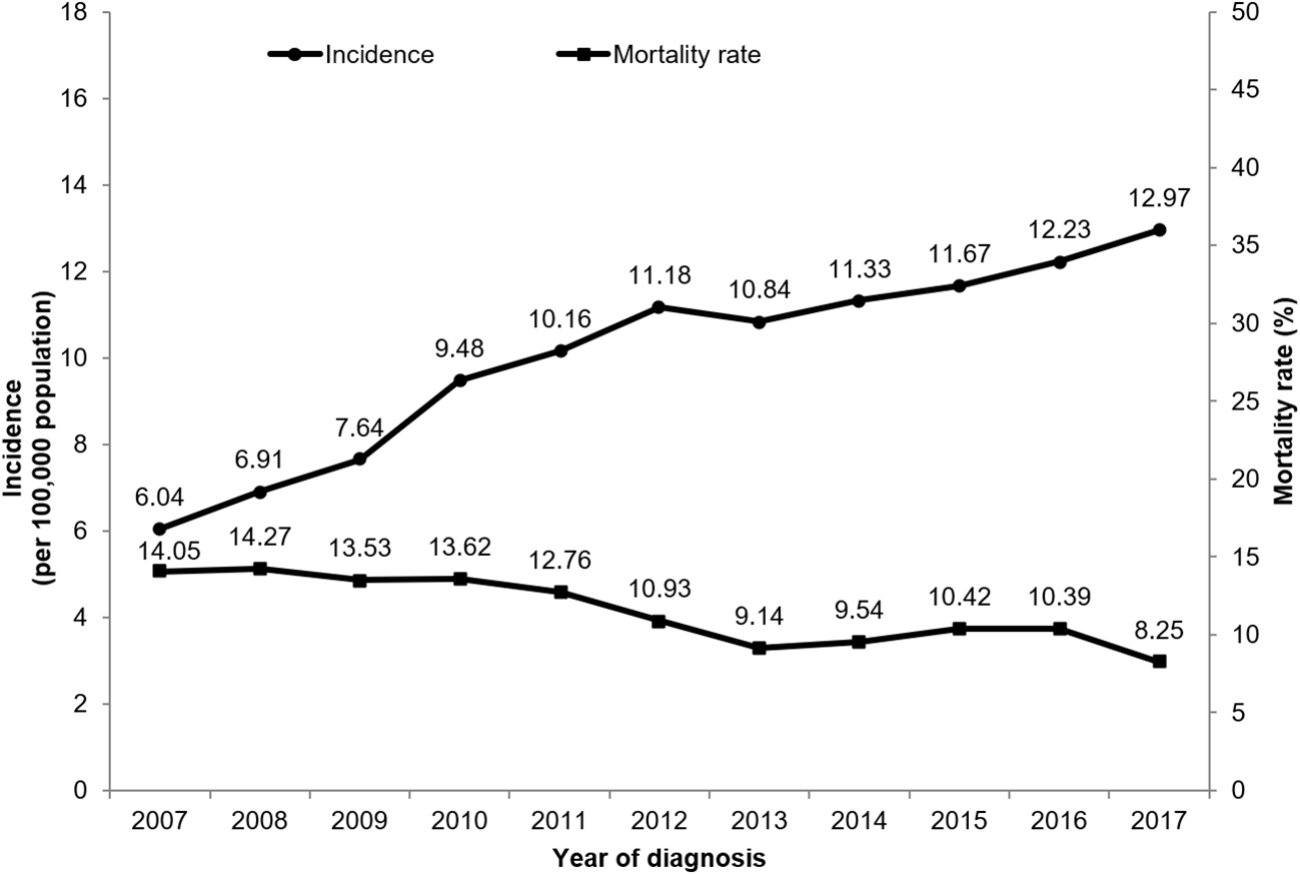
The incidence rate of EO-CRC and the one-year mortality rate of enrolled EO-CRC patients from 2007 to 2017.

The incidence of EO-CRC significantly increased from 6.04 per 100,000 persons in 2007 to 12.97 per 100,000 persons in 2017, and the mortality rate of enrolled EO-CRC patients decreased from 14.05% in 2007 to 8.25% in 2017 (**Figure 2**).

A total of 12,050 patients with EO-CRC, including 6,325 (52.49%) men and 5,725 (47.51%) women, were included in this study (**Table 1**). The mean patient age was 42.40 ± 5.80 years, and 72.45% of patients were 40-49 years of age. Approximately two-thirds of the patients (67.54%) were diagnosed with late-stage EO-CRC (clinical stages III or IV). The overall five-year mortality rate was 32.66%, and the cancer-specific mortality rate was 30.29%.

**Table 1:** The characteristics of EO-CRC patients.

	Overall (N=12050)	Non-survivors (N=3936)	Survivors (N=8114)	p-value
Gender				
**Male**	6325	2057(32.52)	4268(67.48)	0.7265
**Female**	5725	1879(32.82)	3846(67.18)	
**Age**	42.40 ± 5.80	42.08 ± 6.04	42.56 ± 5.68	<0.0001
Age group				
**<30**	394	153(38.83)	241(61.17)	0.0003
**30-39**	2926	1016(34.72)	1910(65.28)	
**40-49**	8730	2767(31.70)	5963(68.30)	
Clinical stage				
**I**	2086	145(6.95)	1941(93.05)	<0.0001
**II**	1826	270(14.79)	1556(85.21)	
**III**	4660	872(18.71)	3788(81.29)	
**IV**	3478	2649(76.16)	829(23.84)	
Pathological stage				
**I**	1672	64(3.83)	1608(96.17)	<0.0001
**II**	2296	162(7.06)	2134(92.94)	
**III**	3451	671(19.44)	2780(80.56)	
**IV**	2596	1889(72.77)	707(27.23)	
**0 or Miss**	2035	1150(56.51)	885(43.49)	
Treatment				
**Operation**	10326	2631(25.48)	7695(74.52)	<0.0001
**Radiotherapy**	2407	850(35.31)	1557(64.69)	0.0019
**Chemotherapy**	9018	3333(36.96)	5685(63.04)	<0.0001
Cancer site				
**Colon**	6877	2400(34.90)	4477(65.10)	<0.0001
**Rectal**	5173	1536(29.69)	3637(70.31)	
CCI group				
**0**	7167	1968(27.46)	5199(72.54)	<0.0001
**1-2**	2725	765(28.07)	1960(71.93)	
**>=3**	2158	1203(55.75)	955(44.25)	
Comorbidity				
**Congestive heart failure**	68	22(32.35)	46(67.65)	0.9563
**Peripheral vascular disease**	36	11(30.56)	25(69.44)	0.7871
**Cerebrovascular disease**	92	46(50.00)	46(50.00)	0.0004
**Chronic pulmonary disease**	205	70(34.15)	135(65.85)	0.6480
**Liver disease**	824	319(38.71)	505(61.29)	0.0001
**Chronic kidney disease**	82	34(41.46)	48(58.54)	0.0882
**Diabetes mellitus**	704	259(36.79)	445(63.21)	0.0161
**Hyperlipidemia**	657	178(27.09)	479(72.91)	0.0017
**Hypertension**	1140	361(31.67)	779(68.33)	0.4505

The overall five-year mortality rate was 101.50 per 1000 person year, and the cancer-specific mortality rate was 94.12 per 1000 person year.

Patients < 30 years of age had a higher non-survivor rate (38.83%) than patients aged 30-39 years (34.72%) and those aged 40-49 years (31.70%; p=0.0003). Patients with clinical stage IV EO-CRC had the highest non-survivor rate (p<0.0001). Patients who underwent treatment also show a significant difference between survivors and non-survivors compared with those who did not undergo treatment. Patients with a CCI score ≥ 3 had a significantly higher non-survivor rate (55.75%) than other CCI groups (p<.0001). Patients with a history of cerebrovascular disease, liver disease, diabetes mellitus, or hyperlipidemia had significantly higher percentages of non-survivors than patients without these comorbidities.

Patients aged <30 years (hazard ratio (HR): 1.19; 95% confidence interval (CI): 1.01-1.40; p=0.0424) and those aged 30-39 years (HR: 1.12; 95% CI: 1.05-1.21; p=0.0017) had a significantly higher overall mortality risk than those aged 40-49 years. Patients with clinical stage IV had a significantly higher mortality risk than those with clinical stage I EO-CRC (HR: 15.67; 95% CI: 13.10-19.75; p<0.0001). Patients who underwent surgical treatment for EO-CRC had a lower mortality risk than those who did not (HR: 0.33; 95% CI: 0.31-0.36; p<0.0001). Patients who underwent chemotherapy had a higher mortality risk than those who did not (HR: 1.11; 95% CI: 1.01-1.22; p=0.0038). Patients with higher CCI scores (CCI score ≥3) had a higher mortality risk than those without comorbidities. Patients with the cerebrovascular disease had a higher mortality risk than those without when adjusted for confounding factors (HR: 1.68; 95% CI: 1.25-2.28, p=0.0007). The cancer-specific mortality risks are similar to those of overall mortality. Patients aged <40 years, those with clinical stage II, III, or IV EO-CRC, those who underwent chemotherapy, those with a CCI score ≥3, and those with cerebrovascular disease all had a higher risk of cancer-specific mortality. Patients who underwent surgical treatment for EO-CRC had a significantly lower mortality risk (**Table 2**).

**Table 2:** The risk of overall and cancer-specific 5-year mortality among EO-CRC patients.

	Overall mortality				Cancer-specific mortality				
	Crude	p-value	AHR	p-value	Crude	p-value	AHR	p-value	
Gender									
Male	1.00(0.94-1.06)	0.9508	1.06(0.99-1.13)	0.0855	0.97(0.91-1.04)	0.3829	1.04(0.97-1.11)	0.2887	
Female	Ref.		Ref.		Ref.		Ref.		
Age group									
<30	1.27(1.08-1.49)	0.0040	1.19(1.01-1.40)	0.0424	1.26(1.06-1.49)	0.0085	1.17(0.98-1.38)	0.0801	
30-39	1.12(1.04-1.20)	0.0023	1.12(1.05-1.21)	0.0017	1.17(1.08-1.26)	<0.0001	1.17(1.08-1.26)	<0.0001	
40-49	Ref.		Ref.		Ref.		Ref.		
Clinical stage									
I	Ref.		Ref.		Ref.		Ref.		
II	2.12(1.73-2.59)	<0.0001	1.98(1.61-2.43)	<0.0001	2.70(2.14-3.41)	<0.0001	2.50(1.97-3.16)	<0.0001	
III	2.94(2.46-3.50)	<0.0001	2.66(2.22-3.20)	<0.0001	3.87(3.14-4.75)	<0.0001	3.45(2.79-4.27)	<0.0001	
IV	22.74(19.23-26.90)	<0.0001	15.67(13.10-18.75)	<0.0001	30.96(25.36-37.79)	<0.0001	20.95(16.98-25.85)	<0.0001	
Treatment									
OP (ref.: no OP)	0.17(0.16-0.19)	<0.0001	0.33(0.31-0.36)	<0.0001	0.17(0.16-0.18)	<0.0001	0.33(0.31-0.36)	<0.0001	
RT (ref.: no RT)	1.09(1.01-1.18)	0.0266	1.05(0.97-1.14)	0.1969	1.10(1.02-1.19)	0.0142	1.05(0.97-1.14)	0.2168	
CT (ref.: no CT)	2.01(1.85-2.19)	<0.0001	1.11(1.01-1.22)	0.0328	2.18(1.98-2.39)	<0.0001	1.16(1.04-1.28)	0.0057	
CCI group									
0	Ref.		Ref.		Ref.		Ref.		
1-2	1.02(0.94-1.11)	0.6563	1.10(1.00-1.20)	0.0414	0.99(0.91-1.08)	0.8919	1.09(0.99-1.19)	0.0836	
>=3	2.66(2.48-2.86)	<0.0001	1.57(1.45-1.69)	<0.0001	2.61(2.43-2.82)	<0.0001	1.54(1.42-1.65)	<0.0001	
Comorbidity									
CHF (ref.: no CHF)	1.04(0.68-1.58)	0.8687	1.03(0.67-1.58)	0.8912	0.81(0.50-1.33)	0.4026	0.83(0.51-1.38)	0.4878	
PVD (ref.: no PVD)	0.96(0.53-1.74)	0.8952	1.03(0.57-1.88)	0.9114	1.04(0.57-1.87)	0.9049	1.14(0.63-2.08)	0.6616	
CVD (ref.: no CVD)	1.87(1.40-2.50)	<0.0001	1.68(1.25-2.28)	0.0007	1.66(1.21-2.29)	0.0018	1.54(1.11-2.14)	0.0102	
CPD (ref.: no CPD)	1.17(0.92-1.48)	0.1967	1.06(0.84-1.35)	0.6140	1.08(0.84-1.39)	0.5623	0.99(0.77-1.29)	0.9545	
Liver (ref.: no Liver)	1.29(1.15-1.44)	<0.0001	1.10(0.97-1.25)	0.1508	1.17(1.04-1.33)	0.0107	1.05(0.92-1.20)	0.4617	
CKD (ref.: no CKD)	1.51(1.07-2.11)	0.0177	0.98(0.68-1.41)	0.9159	0.95(0.61-1.48)	0.8268	0.65(0.41-1.03)	0.0693	
DM (ref.: no DM)	1.17(1.04-1.33)	0.0126	0.96(0.83-1.11)	0.5692	1.15(1.01-1.32)	0.0354	0.97(0.84-1.14)	0.7386	
HPL (ref.: no HPL)	0.82(0.70-0.95)	0.0080	0.87(0.73-1.03)	0.0957	0.81(0.69-0.95)	0.0085	0.89(0.75-1.07)	0.2159	
HTN (ref.: no HTN)	0.96(0.86-1.07)	0.4967	1.00(0.89-1.12)	0.9764	0.93(0.83-1.04)	0.2034	1.00(0.89-1.13)	0.9837	

^1^ Adjusted for gender, age groups (<30, 30-39, and 40-49), clinical stage, treatment (operation, radiotherapy, and chemotherapy), CCI groups (0, 1-2, and >=3), comorbidities (congestive heart failure, peripheral vascular disease, cerebrovascular disease, chronic pulmonary disease, liver disease, chronic kidney disease, diabetes mellitus, hyperlipidemia, and hypertension).

^2^ OP, Operation; RT, Radiotherapy; CT, Chemotherapy, CHF: congestive heart failure, PVD: peripheral vascular disease; CVD, cerebrovascular disease; CPD, chronic pulmonary disease; Liver, liver disease; CKD, chronic kidney disease; DM, diabetes mellitus; HPL, hyperlipidemia; HTN; hypertension.

Both male and female patients with cerebrovascular disease had a significantly higher overall mortality risk compared with those without (males: HR: 1.61; 95% CI: 1.11-2.34; p=0.0128; females: HR: 1.69; 95% CI: 1.00-2.86; p=0.0485) (**Table 3**). Patients aged 40-49 years with cerebrovascular disease had a significantly higher risk of mortality than patients aged 40-49 years without the cerebrovascular disease (HR: 1.63; 95% CI: 1.17-2.27; p=0.0037). Patients with cerebrovascular disease and early clinical stage EO-CRC had a higher risk of overall mortality than patients without (HR: 3.58; 95% CI: 1.84-6.96; p=0.0002), as did patients with cerebrovascular disease and late clinical-stage EO-CRC (HR: 1.49; 95% CI: 1.06-2.09; p=0.0221). Patients with chronic kidney disease and early clinical stage EO-CRC had a higher mortality risk than patients without chronic kidney disease (HR: 2.31; 95% CI: 1.08-4.96; p=0.0138). However, patients with hyperlipidemia and early clinical stage EO-CRC had a lower mortality risk than patients without hyperlipidemia (HR: 0.44; 95% CI: 0.26-0.76; p=0.0027). Patients who underwent radiotherapy had a higher overall mortality risk when paired with cerebrovascular disease CVD (HR: 2.90; 95% CI: 1.41-5.96; p=0.0037) or liver disease (HR: 1.38; 95% CI: 1.04-1.85; p=0.0285) than those without these comorbidities.

**Table 3:** The subgroup analysis of 5-year overall mortality risk among EO-CRC patients of different sex, age, clinical stages, and treatment types.

	Male		Female		<30		30-39		40-49	
Comorbidity ^2^	AHR^1^	p-value	AHR^1^	p-value	AHR^1^	p-value	AHR^1^	p-value	AHR^1^	p-value
CHF (ref.: no CHF)	1.00(0.57-1.74)	0.9860	1.00(0.51-1.95)	0.9927	NA		0.94(0.23-0.89)	0.9340	1.07(0.68-1.68)	0.7581
PVD (ref.: no PVD)	0.96(0.40-2.31)	0.9201	1.14(0.51-2.57)	0.7528	NA		1.17(0.37-3.75)	0.7867	0.98(0.49-1.97)	0.9517
CVD (ref.: no CVD)	1.61(1.11-2.34)	0.0128	1.69(1.00-2.86)	0.0485	NA		2.11(0.87-4.56)	0.0581	1.63(1.17-2.27)	0.0037
CPD (ref.: no CPD)	0.95(0.64-1.40)	0.7960	1.18(0.86-1.61)	0.3000	0.89(0.13-5.89)	0.9010	0.68(0.39-1.19)	0.1775	1.23(0.94-1.61)	0.1320
Liver (ref.: no Liver)	1.16(0.99-1.35)	0.0764	0.97(0.80-1.20)	0.8082	0.55(0.16-1.89)	0.3393	1.07(0.83-1.38)	0.6074	1.10(0.95-1.28)	0.1906
CKD (ref.: no CKD)	0.87(0.57-1.34)	0.5376	1.26(0.64-2.51)	0.5073	NA		0.78(0.19-3.22)	0.7336	1.00(0.69-1.46)	0.9994
DM (ref.: no DM)	0.91(0.76-1.09)	0.3116	1.03(0.80-1.32)	0.8419	NA		1.15(0.76-1.73)	0.5049	0.93(0.79-1.09)	0.3430
HPL (ref.: no HPL)	0.86(0.70-1.06)	0.1501	0.85(0.63-1.15)	0.2835	1.34(0.31-5.79)	0.6955	1.04(0.64-1.68)	0.8764	0.84(0.70-1.01)	0.0623
HTN (ref.: no HTN)	1.03(0.88-1.20)	0.7064	0.95(0.78-1.16)	0.6184	1.95(0.69-5.46)	0.2053	0.82(0.58-1.15)	0.2495	1.02(0.90-1.16)	0.7472
	Early stage		Late stage		Operation		Radiotherapy		Chemotherapy	
Comorbidity^2^	AHR^1^	p-value	AHR^1^	p-value	AHR^1^	p-value	p-value	p-value	AHR^1^	p-value
CHF (ref.: no CHF)	1.62(0.74-3.54)	0.2275	0.85(0.51-1.42)	0.3847	1.12(0.65-1.95)	0.6805	0.55(0.22-1.37)	0.1979	0.82(0.49-1.38)	0.4573
PVD (ref.: no PVD)	NA		1.23(0.68-2.24)	0.4982	1.04(0.52-2.10)	0.9070	1.49(0.55-4.05)	0.4368	1.43(0.76-2.68)	0.2639
CVD (ref.: no CVD)	3.58(1.84-6.96)	0.0002	1.49(1.06-2.09)	0.0221	1.37(0.92-2.02)	0.1184	2.90(1.41-5.96)	0.0037	1.35(0.94-1.93)	0.1055
CPD (ref.: no CPD)	0.68(0.35-1.30)	0.2389	1.09(0.84-1.42)	0.5005	1.00(0.72-1.38)	0.9813	1.22(0.72-2.08)	0.4568	0.91(0.69-1.20)	0.5197
Liver (ref.: no Liver)	1.10(0.78-1.54)	0.6020	1.06(0.92-1.21)	0.4218	1.02(0.87-1.19)	0.8435	1.38(1.04-1.85)	0.0285	0.99(0.86-1.14)	0.8898
CKD (ref.: no CKD)	2.31(1.08-4.96)	0.0138	0.84(0.55-1.27)	0.4119	1.20(0.77-1.89)	0.4236	1.22(0.72-2.08)	0.4568	0.75(0.47-1.21)	0.2422
DM (ref.: no DM)	1.00(0.66-1.52)	0.9960	0.93(0.80-1.09)	0.3814	0.99(0.83-1.18)	0.8722	0.96(0.69-1.33)	0.8160	0.99(0.84-1.17)	0.8925
HPL (ref.: no HPL)	0.44(0.26-0.76)	0.0027	0.95(0.80-1.14)	0.5882	0.88(0.72-1.07)	0.2020	0.72(0.48-1.08)	0.1160	0.85(0.71-1.03)	0.0969
HTN (ref.: no HTN)	0.79(0.55-1.14)	0.2066	1.01(0.89-1.14)	0.9109	0.95(0.82-1.10)	0.4877	0.92(0.71-1.20)	0.5422	1.02(0.90-1.16)	0.7376

^1^Adjusted for gender, age groups (<30, 30-39, and 40-49), clinical stage, treatment (operation, radiotherapy, and chemotherapy), CCI groups (0, 1-2, and >=3), comorbidities (congestive heart failure, peripheral vascular disease, cerebrovascular disease, chronic pulmonary disease, liver disease, chronic kidney disease, diabetes mellitus, hyperlipidemia, and hypertension).

^2^CHF, congestive heart failure; PVD, peripheral vascular disease; CVD, cerebrovascular disease; CPD, chronic pulmonary disease; Liver, liver disease; CKD, chronic kidney disease; DM, diabetes mellitus; HPL, hyperlipidemia; HTN, hypertension; NA, Not Applicable.

Male patients with cerebrovascular disease had a higher cancer-specific mortality risk than those without the cerebrovascular disease (HR: 1.59; 95% CI: 1.07-2.37; p=0.0206) (**Table 4**). Male patients with chronic kidney disease had a lower cancer-specific mortality risk than those without chronic kidney disease (HR: 0.54; 95% CI: 0.31-0.96; p=0.0357). Among patients <30 years of age, those with hypertension had a higher cancer-specific mortality risk than those without hypertension (HR: 2.53; 95% CI: 1.01-6.32; p=0.0471). Among patients aged 30-39 years, those with cerebrovascular disease had a higher cancer-specific mortality risk than those without the cerebrovascular disease (HR: 2.20; 95% CI: 1.02-4.76; p=0.0451). Patients with hyperlipidemia and early clinical stage EO-CRC had a lower mortality risk than those without hyperlipidemia (HR: 0.47; 95% CI: 0.26-0.85; p=0.0121). Patients with cerebrovascular disease and early clinical stage EO-CRC had a higher cancer-specific mortality risk than those without the cerebrovascular disease (HR: 3.30; 95% CI: 1.50-7.27; p=0.0031). Patients with chronic kidney disease and late clinical-stage EO-CRC had a lower risk of mortality than those without chronic kidney disease (HR: 0.54; 95% CI: 0.32-0.92; p=0.0226). Among patients undergoing radiotherapy, those with cerebrovascular disease had a higher cancer-specific mortality risk than those without the cerebrovascular disease (HR: 2.73; 95% CI: 1.27-5.89; p=0.0102). Among patients undergoing chemotherapy, those with chronic kidney disease had a lower cancer-specific mortality risk than those without chronic kidney disease (HR: 0.52; 95% CI: 0.29-0.93; p=0.0281). In addition, the association between different treatment types and CCI is presented in **Supplemental Table 2**.

**Table 4:** The subgroup analysis of 5-year cancer-specific mortality risk among EO-CRC patients of different sex, age, clinical stages, and treatment types.

	Male		Female		<30		30-39		40-49	
Comorbidity^2^	AHR^1^	p-value	AHR^1^	p-value	AHR^1^	p-value	AHR^1^	p-value	AHR^1^	p-value
CHF (ref.: no CHF)	0.77(0.40-1.51)	0.4534	0.88(0.41-1.87)	0.7300	NA		0.47(0.06-3.39)	0.4510	0.91(0.55-1.54)	0.7346
PVD (ref.: no PVD)	1.05(0.43-2.53)	0.9220	1.28(0.57-2.88)	0.5571	NA		1.24(0.39-3.97)	0.7173	1.10(0.55-2.21)	0.7903
CVD (ref.: no CVD)	1.59(1.07-2.37)	0.0206	1.30(0.70-2.42)	0.4078	NA		2.20(1.02-4.76)	0.0451	1.45(1.00-2.10)	0.0502
CPD (ref.: no CPD)	0.90(0.59-1.38)	0.6338	1.08(0.77-1.51)	0.6486	1.67(0.22-12.72)	0.6188	0.73(0.41-1.27)	0.2615	1.12(0.83-1.50)	0.4709
Liver (ref.: no Liver)	1.10(0.93-1.31)	0.2647	0.95(0.76-1.19)	0.6598	0.55(0.13-2.29)	0.4113	1.03(0.80-1.34)	0.8030	1.05(0.90-1.23)	0.5528
CKD (ref.: no CKD)	0.54(0.31-0.96)	0.0357	1.06(0.48-2.36)	0.8859	NA		0.88(0.21-3.62)	0.8583	0.65(0.40-1.05)	0.0788
DM (ref.: no DM)	0.95(0.79-1.15)	0.5714	1.01(0.78-1.32)	0.9227	NA		1.10(0.72-1.67)	0.6722	0.95(0.80-1.12)	0.5476
HPL (ref.: no HPL)	0.92(0.74-1.14)	0.4284	0.81(0.59-1.12)	0.2101	1.33(0.31-5.69)	0.7022	1.12(0.69-1.81)	0.6454	0.86(0.71-1.05)	0.1355
HTN (ref.: no HTN)	1.03(0.88-1.21)	0.7121	0.95(0.78-1.17)	0.6420	2.53(1.01-6.32)	0.0471	0.84(0.60-1.19)	0.3274	1.02(0.89-1.17)	0.7533
	Early stage		Late stage		Operation		Radiotherapy		Chemotherapy	
Comorbidity^2^	AHR^1^	p-value	AHR^1^	p-value	AHR^1^	p-value	AHR^1^	p-value	AHR^1^	p-value
CHF (ref.: no CHF)	0.62(0.15-2.54)	0.5050	0.87(0.51-1.48)	0.6060	0.79(0.39-1.59)	0.5116	0.45(0.16-1.25)	0.1255	0.77(0.45-1.35)	0.3627
PVD (ref.: no PVD)	NA		1.33(0.73-2.42)	0.3469	1.16(0.58-2.34)	0.6766	1.62(0.59-4.42)	0.3474	1.55(0.82-2.90)	0.1748
CVD (ref.: no CVD)	3.30(1.50-7.27)	0.0031	1.38(0.96-1.98)	0.0861	1.24(0.81-1.92)	0.3231	2.73(1.27-5.89)	0.0102	1.31(0.90-1.92)	0.1595
CPD (ref.: no CPD)	0.69(0.34-1.43)	0.3195	1.00(0.76-1.33)	0.9849	0.93(0.66-1.33)	0.6938	1.35(0.79-2.30)	0.2667	0.86(0.64-1.16)	0.3206
Liver (ref.: no Liver)	0.89(0.59-1.35)	0.5874	1.05(0.91-1.21)	0.4745	0.95(0.80-1.13)	0.5540	1.27(0.93-1.73)	0.1361	0.97(0.83-1.12)	0.6419
CKD (ref.: no CKD)	2.09(0.77-5.70)	0.1495	0.54(0.32-0.92)	0.0226	0.77(0.43-1.40)	0.3949	0.32(0.04-2.35)	0.2626	0.52(0.29-0.93)	0.0281
DM (ref.: no DM)	1.17(0.73-1.87)	0.5071	0.94(0.80-1.11)	0.4786	0.99(0.82-1.19)	0.9254	1.01(0.72-1.40)	0.9705	1.00(0.84-1.18)	0.9535
HPL (ref.: no HPL)	0.47(0.26-0.85)	0.0121	0.97(0.80-1.16)	0.7061	0.94(0.77-1.16)	0.5827	0.76(0.50-1.14)	0.1847	0.87(0.71-1.06)	0.1543
HTN (ref.: no HTN)	0.80(0.52-1.21)	0.2859	1.01(0.89-1.15)	0.8899	0.95(0.82-1.11)	0.5095	0.96(0.73-1.25)	0.7591	1.03(0.90-1.18)	0.6511

^1^Adjusted for gender, age groups (<30, 30-39, and 40-49), clinical stage, treatment (operation, radiotherapy, and chemotherapy), CCI groups (0, 1-2, and >=3), comorbidities (congestive heart failure, peripheral vascular disease, cerebrovascular disease, chronic pulmonary disease, liver disease, chronic kidney disease, diabetes mellitus, hyperlipidemia, and hypertension).

^2^CHF, congestive heart failure; PVD, peripheral vascular disease; CVD, cerebrovascular disease; CPD, chronic pulmonary disease; Liver, liver disease; CKD ,chronic kidney disease; DM, diabetes mellitus; HPL, hyperlipidemia; HTN, hypertension; NA, Not Applicable.

## 4 Discussion

The incidence of EO-CRC in Taiwan increased from 2007 to 2017. In our current study, we found that patients with CVD had a higher mortality risk (HR: 1.68; 95% CI: 1.25-2.28; p=0.0007). After subgroup analyses based on age, sex, clinical stage, and treatment type, patients with CVD still had a higher overall mortality risk than those without CVD, except for patients undergoing surgery and chemotherapy. Patients with chronic kidney disease had a higher mortality risk in the early clinical stages (HR: 2.31; 95% C.I.: 1.08-4.96; p=0.0138). Male patients, those with early cancer stage, those undergoing radiotherapy, and those aged 30-39 years or 40-49 years had higher mortality risks than others.

While the exact causes of the increased incidence of EO-CRC are unclear, several possible explanations for the increased incidence of EO-CRC exist. As diet and lifestyle play an important role in the development of CRC, individuals who consume high amounts of red meat, processed meat, and foods high in calories, fat, sugar, and processed carbohydrates become overweight or obese, which is also associated with an increased risk of CRC (25). Shih et al. (26) found that 23.92% of female school children between 11-12 years of age were overweight (25 < BMI ≤ 30), and 9.83% were obese (BMI >30), while 27.08% of boys aged 11-12 years were overweight and 20.35% were obese. Obesity is a risk factor for CRC and has been proven. It has been controversial whether body mass index was a prognostic role in determining the prognosis of patients suffering from CRC. Fanipakdel et al. retrospectively identified 920 patients with non-metastatic CRC and found no statistically significant difference between BMI and mortality (27). Chang et al. conducted three nutrition and health surveys in Taiwan from 1993–1996, 2005–2008, and 2013–2014 (28). The results of these surveys indicated that 80% of the population in Taiwan was <50 years of age and that the prevalence of morbid obesity (BMI ≥35 kg/m (2)) and obesity (BMI ≥27 kg/m (2)) both increased from the first study period to the last. Patients with morbid obesity tended to have lower levels of physical activity in these surveys. Furthermore, patients with morbid obesity consumed more red meat, processed meat products, and sugar-sweetened beverages and lower amounts of fresh fruits, nuts, and dairy products than individuals with normal weight. In Taiwan, the prevalence of obesity increased from 11.8% in 1993-1996 to 17.9% in 2005-2008 and 22.0% in 2013–2014. The prevalence of obesity indicates that the diet of the Taiwanese population has transitioned to one that includes more processed foods high in fats and sugar and calorie-dense foods and beverages (29). These changes lead to obesity and nutrition-related diet-related diseases (30). The rapid growth of international grocery chains and fast-food restaurants leads to food consumption patterns that are similar to those of a Western diet in Asian countries (31).

The underappreciation of the increasing risk of CRC among young adults may also contribute to the increased incidence of EO-CRC as nonspecific symptoms, such as changes in bowel habits, rectal bleeding with bright red blood, blood in the stool, cramping or abdominal pain, and weight loss may be overlooked. Young adults have lower rates of healthcare system utilization than older adults. In 2012, 27% of young American adults (aged 19-34 years) did not have health insurance (32). In 2017, 84.6% of the uninsured population in the United States was 19-64 years old, including one-fourth who were 26-34 years old and one-fifth who were 34-44 years old (33). Taiwan launched a single-payer National Health Insurance program on March 1, 1995, with 99.9% of Taiwan’s population enrolled (34). There are fewer uninsured young adults in Taiwan. Barriers to optimal health care for young adults have been reported (35). Young adults have poor health literacy, little knowledge regarding how to access the medical system and identify providers in the community, and tend to feel embarrassed during doctor visits. In addition, disease presentations in young adults may vary from those in older patients. Physicians who treat diseases that commonly affect older adults, such as cancer, may be less aware of the disease processes in young adults. Studies regarding EO-CRC and oncology training for physicians have emphasized the importance of understanding tumor biology, revealing that the manifestation, epidemiology, and biology of many types of cancer are different in young and old patients (36).

The age of regular CRC screening may also contribute to the increased incidence of EO-CRC. A systematic review confirmed that fecal occult blood test screening improves the survival rate of CRC (37). In 2021, the US cancer screening recommendations for CRC suggested screening adults aged 45-49 years (38) based on the increased incidence of CRC, disease burden, and the assumption that this screening age would have more benefits than screening patients >50 years of age (39). Although most organizations screen adults with an average risk of CRC at 50 years of age, young adults with an increased risk of CRC must undergo more frequent or earlier screening. In our study, EO-CRC patients aged 30-39 years had a higher overall mortality risk than those aged 40-49 years. In addition, cerebrovascular disease played a significant role in prognosis. We recommend screening young adults with cerebrovascular disease for CRC.

Comorbidities may positively or negatively affect the prognoses of patients with CRC (40). For example, patients may schedule regular checkups and necessary follow-up visits with health providers due to comorbidities, at which CRC may be diagnosed at an earlier stage. In contrast, signs and symptoms of CRC may be incorrectly regarded as symptoms of pre-existing comorbidities and may delay diagnosis (41). After the diagnosis of CRC, the timing of treatments, treatment course, and clinical outcomes may be affected by comorbidities. Our results also indicated that EO-CRC patients with a CCI score of more than 2 had higher overall and cancer-specific mortality risk than those with a CCI score of 0. Fowler et al. (42) determined the prevalence of comorbidities in patients with CRC via a survey study and found that one-fourth of patients with CRC had one comorbidity, while 17.7-27.6% of patients had more than one comorbidity. The three most prevalent comorbidities in patients with CRC were hypertension, chronic obstructive pulmonary disease, and diabetes. In this study, the three most common comorbidities were a cerebrovascular disease, chronic kidney disease, and liver disease. The differences in comorbidities may be because 80% of the patients with CRC in the previous study were >60 years of age, while the patients in this study were < 50 years old. Erichsen et al. (43) found that the presence of comorbidities increases the mortality risk in elderly patients with CRC, especially in the first year after diagnosis. In this study, EO-CRC patients with a CCI score ≥ 3 had a higher mortality risk than those without comorbidities. After further analysis, the mortality risk of patients with cerebrovascular disease and chronic kidney disease was increased. Therefore, further research is needed to understand the roles of cerebrovascular disease and chronic kidney disease in patients with EO-CRC.

The incidence of EO-CRC has been reported in several countries, including the USA (44). In Australia and China, the incidence of EO-CRC has increased, which may be attributed to the adoption of Western lifestyles and dietary habits (45). More studies are needed to monitor the incidence of EO-CRC and further evaluate the screening procedures. Furthermore, the underlying causes of EO-CRC should be investigated so that young adults at high risk for CRC who would benefit from early screening can be identified. Physicians must also be aware of the increased incidence of EO-CRC and the appropriate management strategies for comorbidities.

The main strength of this study is the results from the longitudinal population database, which could present the full picture of mortality risk from comorbidities in patients with EO-CRC. However, several limitations of the current study need to be acknowledged. First, our data did not offer information on the socioeconomic status and lifestyle behaviors of the patients, which may affect the estimated mortality risk. Second, misclassification bias might have occurred as the claims data may have differences in the definition of the comorbidities based on the diagnostic codes from the international classification of diseases system. However, the misclassification bias of TCR and the National Health Insurance research database has been validated by a previous study (46). Another potential limitation of registry data may be the lack of actual assessment of patients due to patients not continuing treatments; therefore, the main cause of death or the presence of recurrence may be unclear. However, the national cancer registry of Taiwan has been in operation for over 40 years and the completeness of the TCR is approximately 98.4% (22). Since the TCR is linked to the cause-of-death database and the National Health Insurance research database, we were able to follow these patients almost completely, from being diagnosed with cancer via treatments to death. This limitation can be reduced from linked data. Finally, due to the limited administrative registry databases, information on lifestyles, dietary information, family history of CRC, functional status, physical activity, and CRC screening that may be independently associated with risk of mortality has not been comprehensively recorded.

In conclusion, identifying specific risk factors in patients with EO-CRC allows for the risk stratification of a specific target screening group. More studies are needed to determine if the increased incidence of EO-CRC can be reduced and to identify the associations between comorbidities and EO-CRC. The appropriate management of comorbidities is also important in lowering the incidence of EO-CRC.

## Data availability statement

Publicly available datasets were analyzed in this study. This data can be found here: The data sources are the Taiwan Nation Health Insurance Database and Taiwan Cancer Registry. The data are available with the permission from Taiwan Health and Welfare Data Science Centre (https://dep.mohw.gov.tw/DOS/np-2497-113.html, accessed on 06 Jun 2022). Restrictions apply to the availability of these data, which were used under license for this study.

## Ethics statement

The studies involving human participants were reviewed and approved by Research Ethics Committee of Chi Mei Hospital. Written informed consent for participation was not required for this study in accordance with the national legislation and the institutional requirements.

## Author contributions

Conceptualization, S-CY, Y-LS and K-ML and C-HH. Methodology, S-CY, K-ML and C-HH. Formal analysis, Y-CW. Writing—original draft preparation, S-CY, Y-LS and K-ML. Writing—review and editing, Y-LS, K-ML and C-HH. Visualization, Y-CW. Funding acquisition, C-HH and J-JW. All authors contributed to the article and approved the submitted version.
