# Supplementary material for: The mortality risk in patients with early onset colorectal cancer: the role of comorbidities
Source: Front Oncol. 2023 Apr 14;13:1139925. doi: 10.3389/fonc.2023.1139925 (PMC10147394; doi:10.3389/fonc.2023.1139925)
Supplement: Supplementary file 1 [file Table_1.docx]

**Table S1**. International Classification of Diseases, Ninth Revision, Clinical Modification (ICD-9-CM) and Ten Revision, Clinical Modification (ICD-10-CM) Diagnosis for comorbidities.

| **Comorbidity** | **ICD-9-CM** | **ICD-10-CM** |
| --- | --- | --- |
| Congestive heart failure | 428, 425.4-425.9, 398.91, 402.01, 402.11, 402.91, 404.01, 404.03, 404.11, 404.13, 404.91, 404.93 | I09.9, I11.0, I13.0, I13.2, I25.5, I42.0, I425-I429, I43, I50, P29.0 |
| Peripheral vascular disease | 440, 441, 437.3, 443.1-443.9, 471, 093.0, 557.1, 557.9 | I70, I71, I73.1, I738, I73.9, I77.1, I79.0, I79.2, K55.1, K55.8, K55.9, Z95.8, Z95.9 |
| Cerebrovascular disease | 430-438, 362.34 | G45, G46, I60-I69, H34.0 |
| Chronic pulmonary disease | 490-505, 416.8, 416.9, 506.4, 508.1, 508.8 | J40-J47, J60-J67, J68.4, J70.1, J70.3, I27.8, I27.9 |
| Liver disease | 070.6, 070.9, 070.22, 070.23, 070.32, 070.33, 070.44, 070.54, 456.0-456.2, 570, 571, 572.2-572.8, 573.3, 573.4, 573.8, 573.9 | B18, I85.0, I85.9, I86.4, I98.2, K70.0-K70.4, K70.9, K71.1, K71.3-K71.5, K71.7, K72.1, K72.9, K73, K74, K76.0, K76.2-K76.9, Z94.4 |
| Chronic kidney disease | 585 | N18 |
| Diabetes mellitus | 250 | E08-E13 |
| Hyperlipidemia | 272 | E78 |
| Hypertension | 401-405 | I10-I13, I15 |
