# Supplementary material for: The mortality risk in patients with early onset colorectal cancer: the role of comorbidities
Source: Front Oncol. 2023 Apr 14;13:1139925. doi: 10.3389/fonc.2023.1139925 (PMC10147394; doi:10.3389/fonc.2023.1139925)
Supplement: Supplementary file 2 [file Table_2.docx]

**Supplemental Table 2.** The 5-year overall and cancer-specific mortality risk between CCI group and different treatment types among EO-CRC patients

| 5-year overall mortality risk among EO-CRC patients | | | | | | |
| --- | --- | --- | --- | --- | --- | --- |
|  | **Operation** |  | **Radiotherapy** |  | **Chemotherapy** |  |
| CCI group | **AHR^1^** | **p-value** | **AHR^1^** | **p-value** | **AHR^1^** | **p-value** |
| 0 | Ref. |  | Ref. |  | Ref. |  |
| 1-2 | 1.12(1.00-1.24) | 0.0439 | 1.06(0.87-1.30) | 0.5604 | 1.10(1.00-1.21) | 0.0499 |
| >=3 | 1.66(1.51-1.83) | <0.0001 | 1.66(1.39-1.99) | <0.0001 | 1.42(1.31-1.55) | <0.0001 |
| 5-year cancer-specific mortality risk among EO-CRC patients | | | | | | |
|  | **Operation** |  | **Radiotherapy** |  | **Chemotherapy** |  |
| CCI group | **AHR^1^** | **p-value** | **AHR^1^** | **p-value** | **AHR^1^** | **p-value** |
| 0 | Ref. |  | Ref. |  | Ref. |  |
| 1-2 | 1.11(0.99-1.24) | 0.0712 | 1.04(0.84-1.28) | 0.7272 | 1.09(0.99-1.21) | 0.0797 |
| >=3 | 1.62(1.47-1.80) | <0.0001 | 1.64(1.36-1.98) | <0.0001 | 1.42(1.30-1.55) | <0.0001 |
